# Evaluating Depth-Based Human Pose Estimation in Real-World Nursing Home Environments

**DOI:** 10.3390/s26196256

**Published:** 2026-10-02

**Authors:** Rinu Elizabeth Paul, Tanja Schultz

**Affiliations:** Cognitive Systems Lab, University of Bremen, Enrique-Schmidt-Str. 5, 28359 Bremen, Germany

**Keywords:** human pose estimation, depth images, depth sensors, nursing homes, multi-person pose estimation, real-world monitoring

## Abstract

Human pose estimation in real-world indoor environments remains challenging due to privacy concerns, occlusions, varying numbers of people, and noisy sensor data. In this work, we investigate pose estimation from depth images captured over an extended period in a nursing home setting. We explore multiple input representations, including depth-only and silhouette-based representations, as well as depth-based Sobel gradient features. To systematically analyze the impact of model complexity and input representation, we design an experimental framework comparing lightweight Convolutional Neural Networks (CNNs), graph-based models, and deeper architectures, including ResNet and HRNet. Among the evaluated configurations, CNN+GCN with Depth+Sobel input achieved the highest test PCK on the seven-joint baseline, reaching 77.18% in the room-dependent setting and 76.03% in the room-independent setting. Lightweight models generally outperformed higher-capacity architectures on this dataset, but the experiments do not identify the mechanism behind this difference. CNN+LSTM reduced frame-level performance compared to the corresponding frame-wise CNN. Under realistic conditions, performance decreased for partially visible and multi-person scenes. Adding a visibility prediction head produced modest improvements in the evaluated scenarios. Unoccupied-room training reduced spurious pose predictions. The results indicate that lightweight architectures with structural depth representations are promising for privacy-sensitive pose estimation in real-world nursing home environments. However, substantial challenges remain for partial visibility, multi-person scenes, annotation uncertainty for occluded joints, and end-to-end deployment. Computational measurements show low model inference time on the tested hardware but do not establish end-to-end real-time system performance.

## 1. Introduction

Image-based video monitoring has applications across a large range of domains, including security, healthcare, sports, manufacturing, smart cities, and agriculture. Some of the main applications are intrusion detection, defect detection on assembly lines, patient monitoring, traffic flow analysis, and crop health assessment [1,2]. Human pose estimation, which identifies and extracts keypoints from a given image. Keypoints represent major joints such as the head, neck, spine, torso, shoulders, hips, knees, elbows, wrists, toes, fingers, and ankles, forming a human structure. Pose estimation is a fundamental component of several applications, including security, healthcare, and sports. In particular, in applications such as patient monitoring, assisted living, and activity-based human–computer interfaces, reliable pose estimation is crucial, as it underpins understanding of human motion and unusual behavior [3,4].

Human monitoring applications can be implemented using other sensors, such as wearables or smartphones. Some applications yield better results with wearables, as they are worn directly by humans and sensor data can be used to obtain results [5]. However, for older adults, such wearables are uncomfortable and often forgotten [6]. Wearable sensors also require regular charging, maintenance, and correct placement, which can reduce their suitability for long-term, continuous monitoring [7,8]. This requires software changes and updates with sensor versions.

The main advantages of image-based sensors for continuous monitoring include non-invasiveness and the absence of wearable components. Some sensors have a variable field of view, and smartphone drones are limited by battery life. The most commonly used depth sensors in these contexts include time-of-flight (ToF) sensors, structured light systems, and stereo vision cameras [9]. These sensors have technical constraints, including limited range (typically up to 5 m for consumer-grade ToF systems), reduced accuracy in direct sunlight, sensitivity to reflective or dark surfaces, and lower spatial resolution than high-definition RGB cameras [10]. Frame rates are commonly 15 to 30 frames per second, which may limit the temporal resolution for fast actions [11]. In healthcare applications, privacy concerns limit certain use cases [12], such as fall detection and behavior tracking. In privacy-sensitive scenarios, Depth-only sensing can reduce the exposure of identifiable visual appearance compared with conventional RGB video while enabling monitoring [13].

Recent advances in deep learning have improved pose estimation accuracy, particularly with RGB images. However, RGB-based approaches are more sensitive to lighting conditions and raise privacy concerns, limiting their suitability for continuous monitoring in settings such as nursing homes or clinics. Depth sensors, by contrast, offer privacy and are more robust to illumination changes [14]. While depth-based pose estimation generally lags behind RGB approaches in raw accuracy due to reduced spatial detail, limited texture, and susceptibility to noise, the trade-offs in privacy preservation and robustness make it a valuable option for human pose estimation.

In real-world monitoring systems, image-based approaches are often required to support multiple simultaneous tasks, many of which demand rapid, near-real-time responses. In such time-critical multitasking settings, high-capacity image-based deep-learning approaches may be computationally expensive for multi-room, multi-task deployment. This not only raises the number of model parameters but also places substantial demands on memory and processing power. As the number of concurrent tasks increases, these requirements scale rapidly, making such systems expensive and often impractical to deploy in resource-constrained or hardware-limited environments [11]. Deploying AI-driven monitoring systems for tasks such as fall detection, gait, or mobility monitoring in nursing homes is further slowed by reliance on image data, and multi-room setups require simultaneous processing from multiple sensors, further stressing resources [15].

Typical depth images are 640 × 480, 16-bit single-channel data (depth image) [16], while RGB images consist of three channels. Despite their efficiency advantages, depth images introduce additional challenges for learning-based systems. A key challenge is generalization: depth images often contain sensor-specific features, such as unique noise patterns, quantization artifacts, and variations in intrinsic parameters. Depending on how models are trained, they may unintentionally rely on these sensor-specific cues, limiting their ability to generalize across different sensor types, environments, or deployment sites. Rather than capturing underlying human structure or motion patterns, the model might exploit sensor-data idiosyncrasies, reducing performance on novel or unseen data [17].

Possible strategies to mitigate these challenges include data augmentation techniques that simulate sensor variability, domain adaptation methods to align feature distributions between sensors, and sensor-invariant representation learning approaches. Incorporating multi-sensor training datasets and applying regularization can also help promote generalization to a range of real-world scenarios.

### 1.1. Related Work and Research Gap

Researchers have extensively studied human pose estimation from images. Several studies have examined 2D and 3D pose estimation using RGB, RGB-D, and D-only images, as well as motion capture data. Hartmann et al. [18,19] compared several pose estimation models on a single privacy-preserving depth sensor using deep models (e.g., ResNet50), enabling the extraction of joint positions with centimeter-level error despite limited viewpoint information. They also extended their approach to sequence-based pose estimation to smooth the extracted poses. Depth-based pose estimation methods leverage sensors such as Microsoft Kinect to capture 3D structural information of human motion. Datasets such as Human3.6M and NTU RGB+D [20] are collected in indoor environments using one or more depth sensors over extended durations, supporting both single- and multi-person scenarios. Depth-based pose estimation can achieve reasonable accuracy in controlled environments, but is typically validated on structured datasets with limited real-world variability.

HRNet [21] maintains high-resolution representations throughout the network and has been widely used for accurate pose estimation. It is typically evaluated on RGB datasets such as COCO and MPII, collected in controlled and in-the-wild environments using monocular cameras, that involve multi-person scenarios but are primarily limited to short-duration annotated frames. ResNet-based [22,23] architectures have been widely used as backbones for pose estimation due to their strong feature-extraction capabilities. These models are commonly trained on large-scale RGB datasets collected with single cameras in both laboratory and real-world environments, supporting both single- and multi-person pose estimation. OpenPose [24] is a bottom-up multi-person pose estimation framework that detects keypoints and associates them using part affinity fields. It operates on RGB data collected from monocular or multi-camera setups in real-world environments and is widely used for multi-person scenarios in short-duration image datasets. PopNet [25] is a pose estimation network designed for efficient inference in embedded or real-time systems. It has been evaluated on RGB datasets with single-camera setups, typically focusing on single-person or limited multi-person scenarios in controlled environments. RGB-based pose estimation methods are highly accurate and scalable, but rely heavily on well-annotated datasets that do not reflect long-term, noisy, or continuously changing real-world monitoring conditions.

Silhouette-based [26,27] approaches extract binary foreground masks from depth or RGB data to represent human body shape while suppressing background information. These methods are commonly evaluated in controlled laboratory settings using single or multiple sensors and have shown effectiveness for single-person pose estimation. However, performance may degrade in multi-person or occluded scenarios. Ding et al. [28] proposed a silhouette-based method for human pose estimation using iterative algorithms and Euclidean distance measures, demonstrating its effectiveness on controlled image data for single-person motion analysis. Zhao and Lee [29] presented a human silhouette extraction approach based on Hidden Markov Models (HMM), evaluated on image sequences in laboratory settings for foreground segmentation and shape-based analysis. In [30], 2D pose estimation and segmentation were combined to predict human silhouettes under occlusion using lab-recorded RGB images. Silhouette-based methods can simplify pose estimation by reducing visual complexity, but they remain largely constrained to controlled settings and struggle with occlusion and multi-person interactions.

Most state-of-the-art research focuses on RGB-based 2D pose estimation and RGBD- or depth-only-based 3D pose estimation with motion-capture ground truth, mostly using lab-based datasets. Their performance and generalizability to noisy depth data from real-world environments remain less established. Real-world data include scenarios such as unoccupied rooms, single-person scenes with missing joints, and two-person scenarios. Existing approaches are often evaluated under controlled conditions and may not adequately address the combination of noisy depth measurements, partial visibility, multi-person interactions, and unoccupied scenes encountered in long-term nursing home monitoring [31]. Although occlusion handling is widely studied in computer vision, most methods are evaluated in controlled settings. Real-world nursing home monitoring introduces noisy depth data, helping aids, and complex multi-person interactions. Therefore, efficient and robust depth-based pose estimation methods remain needed for real-world healthcare environments.

### 1.2. Objectives and Paper Structure

This paper investigates 2D human pose estimation from depth images collected in realistic healthcare environments, focusing on nursing home monitoring. Human poses are represented by body-joint keypoints such as the head, shoulders, elbows, knees, and ankles. The main objective is to develop pose estimation methods that are accurate, computationally efficient, and robust to conditions encountered in continuous, privacy-preserving monitoring.

To achieve this objective, we systematically evaluate pose estimation models with different input representations and architectural complexities. We first compare high-capacity ResNet18- and HRNet18-based architectures with lightweight CNN models using raw depth, silhouette, and Depth+Sobel representations. Then, we investigate whether explicitly modeling spatial relationships between joints with a Graph Convolutional Network (GCN) or temporal dependencies with an LSTM can improve pose estimation. Model selection considers pose estimation performance and computational requirements such as inference time, memory consumption, complexity, and parameter count to assess suitability for real-time deployment.

Finally, we evaluate the selected approaches under realistic nursing home conditions, including partially visible joints, multiple persons, and unoccupied rooms. To improve robustness, we investigate visibility-aware prediction and explicit supervision for missing joints and empty scenes. The main contributions of this work are:

- A systematic comparison of depth, silhouette, and Depth+Sobel representations across lightweight and high-capacity pose estimation architectures.
- An analysis of spatial graph modeling and temporal modeling for depth-based pose estimation.
- An evaluation of pose estimation robustness under partial visibility, multi-person, and unoccupied-room conditions using realistic nursing home data.
- An evaluation of visibility-aware supervision for handling partially visible joints and unoccupied scenes.
- A computational analysis of model complexity, memory consumption, parameter count, and inference time for practical deployment.

The remainder of the paper is organized as follows. Section 3 explains the evaluation metrics for measuring model performance, followed by Section 2, which describes the dataset and input representations used. Section 4 explains the methodology and results, and Section 5 concludes with a summary and discussion of future work.

## 2. Dataset and Preprocessing

We used our dataset ETAP-Depth Image Dataset (ETAP-DID) in this paper. We collected the data using sensor boxes developed by DHC Digital HealthCare Systems GmbH that integrate stereo vision depth sensors; we obtained an approved ethical proposal and informed consent from the necessary individuals [32]. We collected data from three nursing homes using wall-mounted depth sensors, with one sensor per resident room, providing realistic variations in occupancy, activities, occlusions, and sensor conditions. We collected the dataset for 21 months from three nursing homes using wall-mounted depth sensors, with one sensor per resident room. It contains 77,400 h of 16-bit depth data from 48 residents aged 60 years and older, recorded at 10 fps with a resolution of 640 × 480 pixels. However, we used only the manually annotated subset described in the annotation section to train and evaluate supervised pose estimation.

**Figure 1:**
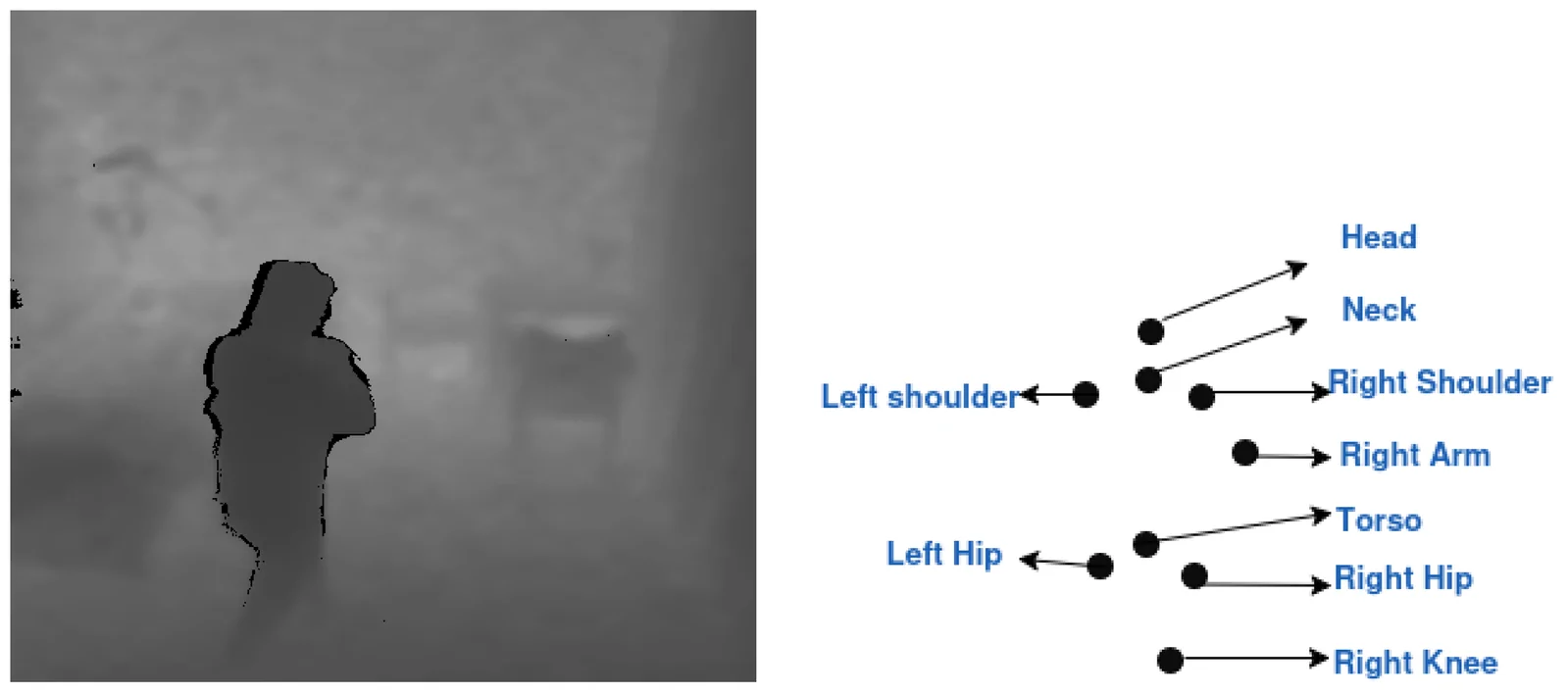
Depth image of a resident from a nursing home. The body keypoint joints labeled in the depth image (**left**) are given on the (**right**).

We mounted sensors at heights between 2.10 m and 2.25 m and tilted them downward between 30° and 40°, based on room dimensions and furnishings, to capture a full-body view, with placement optimized for safety, unobtrusiveness, and visibility. Figure 1 presents a sample frame of a person entering a resident’s room. Each depth image pixel encodes a distance value from the sensor. Image pixels were converted to physical coordinates (millimeters) using the sensor-specific depth scale. We then used these millimeter-based coordinates.

The ETAP-DID includes residents, staff members, and visitors at the nursing homes. Because the ETAP-DID is collected in a real-world environment, it includes various pose scenarios. The ETAP-DID includes several activities, such as walking, standing, sitting, and transition activities, with residents using walking aids. Frames include a single person, two or more people, no person, or a person with fewer visible joints (due to occlusion, sensor field of view, or distance from the sensor).

### 2.1. Sensor Field of View and Coverage

The stereo-vision depth sensor has a field of view of H 75° ± 3°, V 62° ± 1°, and D 89° ± 3°. At a distance *d*, the approximate coverage is given by

(1) $$\mathrm{Width}=2d\tan\left(\frac{\theta_{H}}{2}\right),\;\mathrm{Height}=2d\tan\left(\frac{\theta_{V}}{2}\right)$$

At 2 m, the sensor covers about 3.07 m horizontally and 2.40 m vertically. Mounted above head height and tilted downward, the effective floor coverage is trapezoidal and depends on mounting height and tilt angle. In this study’s configurations, the opposite wall mainly limited the observed room depth rather than the vertical field of view. These calculations justify the selected configuration geometrically, although we did not systematically optimize height and angle.

### 2.2. Data Annotation

We manually annotated 15 h of depth data from 30 occupied unique rooms using the Computer Vision Annotation Tool (CVAT) [33]. For each frame, the skeleton of a resident was annotated with 15 body joint keypoints. To ensure consistency among annotators, we provided proper training and evaluation. We assessed annotation consistency using PCK (see Equation (4)), with average agreement above 85% on the evaluated annotation subset. Annotators labeled all visible joints in each frame using CVAT. For partially visible persons, annotators estimated the most likely location of joints not directly observable from the depth image. Consequently, evaluation of these joints reflects both pose-estimation error and uncertainty in the inferred annotation. We interpret performance on missing or occluded joints as an assessment of clinically relevant pose plausibility under partial visibility, not as equivalent to directly observed joint localization. For better visualization during annotation, we converted the depth data to videos after adjusting contrast and brightness. The body joint keypoints annotated were head, neck, right shoulder, left shoulder, right hip, left hip, right knee, left knee, right ankle, left ankle, right elbow, left elbow, right wrist, left wrist, and torso (e.g., in Figure 1(right)). We considered the torso as the root joint. In this ETAP-DID, 10% of frames featured two people, and the others featured a single person. We annotated frames with no visible person in the room as unoccupied. We split the annotated single-person data into train, validation, and test sets in an 80:10:10 ratio. We added multi-person data only to the validation and test sets to fine-tune the multi-person pose estimation model. We also added 2.5 h of manually annotated unoccupied data to the training set and 30 min to the validation and test sets to train and test the models for unoccupied-room scenarios (see Table 1). Therefore, the total duration of data used with occupied-room and unoccupied-room frames is 18 h. The dataset contains 30 unique rooms, with occupied and unoccupied recordings from each. Rooms are assigned to only one split to prevent overlap between training, validation, and test sets.

### 2.3. Image Preprocessing and Representations

During ETAP-DID preparation, we encoded and processed each frame to remove artifacts and noisy depth values. Pixels with values of zero represented missing data or sensor artifacts, while values above 4000 corresponded to distances greater than four meters, where visibility degrades and depth measurements become unreliable [34]. Because these regions were considered least important for pose estimation, we replaced pixels with values of zero or above 4000 with the mean pixel value computed from valid pixels within the same frame, excluding zeros and values above 4000. We chose the mean because it preserves the overall depth distribution without introducing artificial edges or biasing the data toward extreme values, reducing the risk of the model learning spurious cues from placeholder values. We then normalized depth values using the minimum and maximum values computed across all frames within each room.

#### 2.3.1. Sobel Gradient

**Figure 2:**
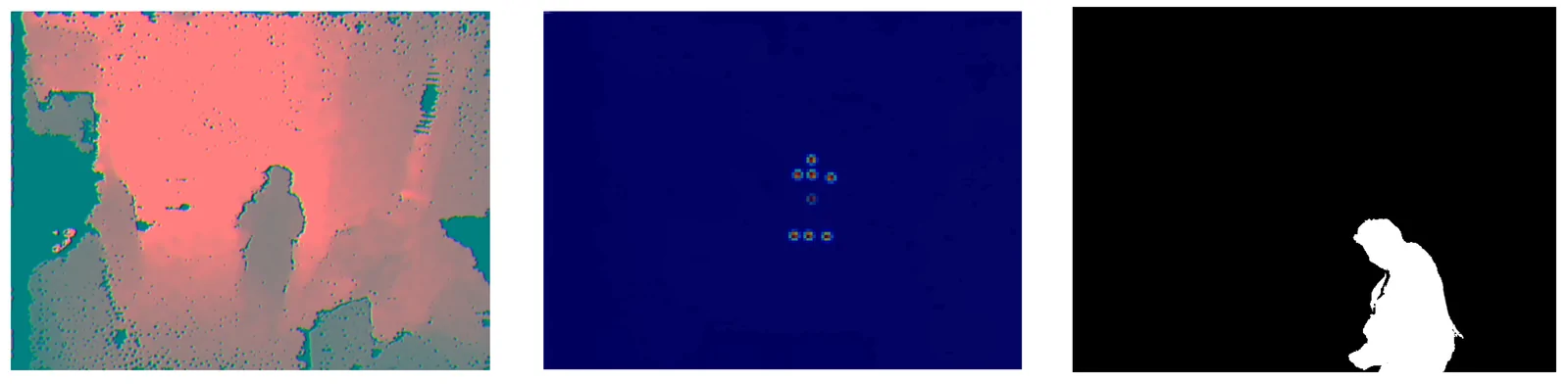
Sobel xy+depth as an RGB image (**left**); combined center and joint heatmaps (**middle**); extracted silhouette (**right**).

The depth images have a single channel, with each pixel denoting the distance from the sensor. To add structural context, since the color channels are missing, we computed vertical and horizontal Sobel gradients for each pixel. We derived Sobel gradients in the x- and y-directions from the depth channel, forming a three-channel input. Sobel *x* and *y*, computed from depth changes, reveal edge variations and improve localization of human body parts [35]. For each depth image, horizontal and vertical gradients were calculated as follows:

$$S_{x}=\left[\begin{array}{ccc} -1 & 0 & +1\\ -2 & 0 & +2\\ -1 & 0 & +1 \end{array}\right]\;S_{y}=\left[\begin{array}{ccc} -1 & -2 & -1\\ 0 & 0 & 0\\ +1 & +2 & +1 \end{array}\right]$$

(2) $$G_{x}=I*S_{x}$$

(3) $$G_{y}=I*S_{y}$$

where *I* denotes the depth image, and $G_{x}$ and $G_{y}$ are the horizontal and vertical gradient maps at each pixel (i,j), which are stacked with the original depth channel to form the final three-channel input. Figure 2(left) shows the combined image, with Sobel *x*,*y*, and depth channels for a depth frame as RGB channels.

#### 2.3.2. Heatmap

For the Convolutional Neural Network (CNN) models (refer to Section 4), we generated heatmaps from the root and ground-truth joint coordinates, which are compared to the estimated heatmaps by the model. We created each heatmap by centering a two-dimensional Gaussian function (a bell-shaped kernel that smoothly spreads values around a point) at the corresponding ground-truth joint position and the skeleton root joint in the image. The Gaussian function assigned higher values near the joint location and gradually decreased toward the periphery, highlighting probable joint areas. This gradual diminution reflects the decreasing likelihood that a joint lies from its ground-truth position while providing a smooth spatial representation around the target location. We used a standard deviation of 2 pixels for the Gaussian kernel, which determined the spread and softness of each peak. We normalized the heatmap intensity values to the range [0, 1]. We used these heatmaps to support the model during pose estimation. The center and joint heatmaps for a skeleton in a depth frame are shown in Figure 2(middle).

#### 2.3.3. Silhouette

Silhouette extraction (binary foreground masks representing the human shape) used image-processing methods such as background subtraction. The approach removed a static background while preserving the moving subject [36]. The process began with far- and floor-pixel elimination [37] using a dataset-specific threshold. We then normalized all values to [0, 1]. Reference-image subtraction [38] was applied with far- and floor-pixel removal and normalization. The reference frame contained only background. We obtained the final silhouettes via connected component labeling [39], which grouped pixels of similar depth and filtered out noise patches based on component size. Figure 2(right) shows the extracted silhouette from a depth image.

## 3. Evaluation Metrics

### 3.1. Percentage of Correct Keypoints

We used the Percentage of Correct Keypoints (PCK), which measures the percentage of joints within a certain distance (typically 15 cm in prior pose estimation research) from the ground truth to evaluate the performance of the pose estimation model [40] given in Equation (4), where $K_{i}$ is the estimated joint keypoint, $J_{i}$ is the ground truth, and N denotes the total number of joint keypoint classes in the evaluation. This metric ranges from 0 to 100%, with higher values indicating better performance.

(4) $$PCK=\frac{100}{N}\sum_{i=1}^{N}\left\{\begin{array}{cc} 1, & \mathrm{if}\;∥K_{i}-J_{i}{∥}_{2}<15\;cm\\ 0, & \mathrm{else} \end{array}\right.$$

### 3.2. Mean Average Precision (mAP)

Mean Average Precision (mAP) given in Equation (5) was used to evaluate the estimated keypoints [41]. For each joint, we calculate Average Precision (AP) using Object Keypoint Similarity (OKS), which measures alignment between predicted and ground-truth keypoints. mAP is obtained by averaging AP across all visible joints and thresholds, summarizing overall detection accuracy. Higher mAP values reflect better keypoint localization.

(5) $$mAP=\frac{1}{N}\sum_{i=1}^{N}AP_{i}$$

For the two-person scenario, we evaluated an HRNet-based configuration with depth input because the lightweight configuration did not reliably separate the two persons in preliminary development. We used a center heatmap to identify person instances and associate predicted joints with individual centers. Since this evaluation uses a different architecture and prediction procedure than the single-person experiments, we interpret the results as a scenario-specific feasibility assessment, not a direct architectural comparison.

For unoccupied-room frames, the primary objective was to suppress spurious pose predictions rather than localize anatomical keypoints. We therefore evaluated predictions based on whether the model correctly produced no person-level pose. This reflects hallucination or false-positive pose suppression, not conventional keypoint localization accuracy. For unoccupied frames, we treated all joints as invisible during evaluation.

## 4. Pose Estimation

We evaluate the proposed pose estimation framework across three settings: (i) model and image representation selection, (ii) temporal modeling, and (iii) robustness under real-world deployment scenarios. We assessed the second and third scenarios using the best-performing model and input representation, allowing us to evaluate the proposed approaches systematically while minimizing redundant experiments.

In the model and input representation experiments, we established a reference performance using a baseline dataset. Although 15 joints were annotated in the ETAP-DID, the baseline dataset used only seven limb joints that were consistently visible. The baseline dataset consisted of 7.5 h of data containing a single person, with all non-limb joints (head, neck, shoulders, torso, and hips) visible, and we used the same data split (80:10:10). In all the experiments, we used a session-independent split so that continuous recordings do not appear in training and testing sets. All the models were trained for 40 epochs using the AdamW optimizer (learning rate = $1\times 10^{-3}$ weight decay = $1\times 10^{-4}$) with a ReduceLROnPlateau scheduler (factor = 0.5, patience = 5), and all experiments were made reproducible by fixing random seeds in PyTorch version 5.12 with deterministic CuDNN settings.

### 4.1. High-Capacity Architectures

We started with evaluated input representations using high-capacity architectures commonly used in prior work. We used two input configurations: depth images only and depth images with Sobel gradients. We evaluated these inputs using two CNN architectures for frame-wise pose estimation: ResNet18 and HRNet18. We chose these for comparison because ResNet18 provides an efficient baseline for feature extraction, while HRNet18 maintains high-resolution representations throughout the network, making it more effective for precise spatial localization in pose estimation. This stage evaluated whether additional structural priors and higher-capacity architectures improved performance beyond lightweight representations. These models estimated the center and joint heatmaps from the input representation; in validation and testing, we then calculated joint positions from these heatmaps. For ResNet and HRNet models, we used the L1 loss between the predicted and target heatmaps since these models primarily focus on improving heatmaps rather than directly predicting joints.

Table 2 summarizes the performance of various input representations and model architectures. HRNet18 consistently outperforms ResNet18, confirming the benefit of preserving high-resolution features. Both models performed worse when using silhouette as input. Both models exhibit reduced generalization when depth images are combined with Sobel features, suggesting that overly dense supervision may introduce noise in pose estimation.

### 4.2. Lightweight Architectures

The low performance and high hardware requirements of high-capacity models led us to explore lightweight architectures on the baseline dataset. As in real-world scenarios, the goal is to have models that are fast and require less hardware. In this stage, we evaluated lightweight input representations using computationally efficient models. The input configurations considered included depth images, silhouette images extracted from depth data, and depth images with Sobel gradient channels. We evaluated these inputs using a simple CNN model for frame-wise pose estimation and a CNN-based feature extractor combined with Graph Convolutional Networks (GCNs), the CNN+GCN model, to incorporate spatial relationships between joints. These models predicted skeletal joint coordinates directly from input representations. We used a Smooth L1 loss ($L_{pose}$), which behaves like L1 loss for large errors and like L2 loss for small errors, making it robust to outliers while maintaining stable convergence. To enforce spatial correctness, we use a differentiable PCK loss ($L_{pck}$), which rewards predictions within a distance threshold of the ground truth and improves structural consistency beyond what direct regression can achieve. The total loss function $L_{total}$ combines both loss terms, with their respective weights as hyperparameters. The final model performance score is computed as $1-L_{total}$.

**Figure 3:**
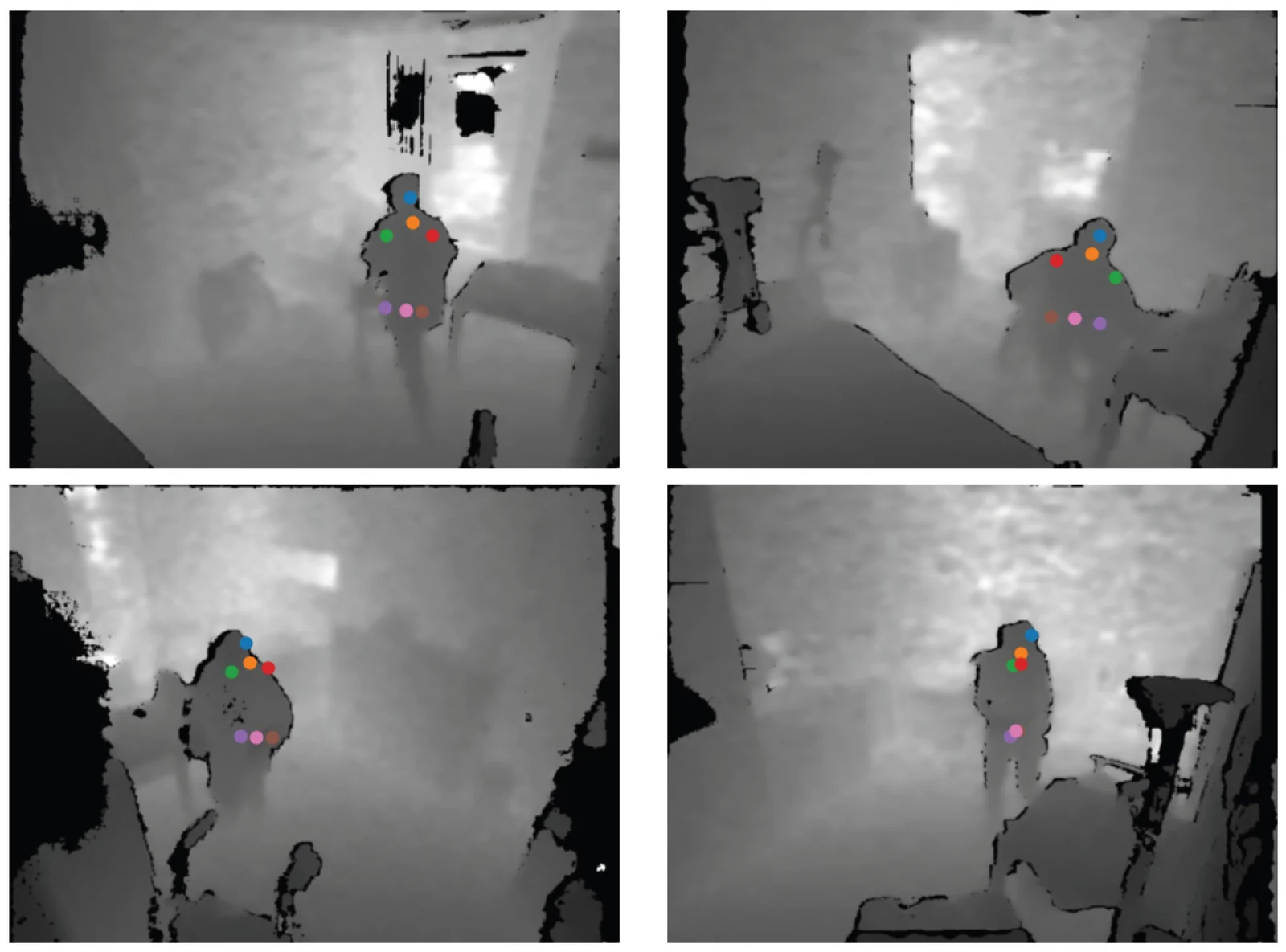
The resultant skeleton joints, as colored dots (baseline), are extracted and overlaid on depth images using CNN+GCN on the Depth+Sobel representation. The **top** row shows examples of best pose estimation results, and the **bottom** row shows examples of incorrect estimation results.

The CNN+GCN variant achieves the best results in this category, with a test performance score of 84.28% and PCK of 77.18%, indicating that edge-aware representations enhance spatial localization. The CNN+GCN model outperformed the vanilla CNN for depth and Depth+Sobel inputs, while the vanilla CNN performed better for silhouette inputs. These results demonstrate the advantage of explicitly modeling joint dependencies through graph-based refinement. The resultant skeleton joints (baseline), extracted using Depth+Sobel with CNN+GCN and overlaid on depth images, are shown in Figure 3. The top images show that the seven extracted keypoints are close to the joint positions in the depth image, whereas the bottom images show that some or most of the extracted keypoints are away from the positions in the corresponding depth image.

For the room-independent evaluation, we obtained test recordings from rooms not used for model training. This setting, therefore, evaluates generalization across previously unseen room environments. Performance decreases across all configurations, as expected, due to reduced spatial consistency. Here, a room is equivalent to a sensor. Depth with Sobel remains the most robust representation, with CNN+GCN achieving an 81.67% performance score and 76.03% PCK on the test set. These results indicate that gradient-enhanced depth representations generalize more effectively across environments than raw depth or silhouette inputs.

We evaluated statistical significance using two paired t-tests and Wilcoxon signed-rank tests over 500 test frames. For each frame, we computed PCK by aggregating joint-level correctness against ground-truth annotations. We used the resulting per-sample PCK values for paired statistical testing. We compared the paired PCK values of the evaluated models on the same test frames. Because the frames come from continuous recordings and may exhibit temporal dependence, interpret the statistical comparisons descriptively rather than as evidence of population-level significance. We emphasize the observed performance differences and effect sizes rather than treating the frame-level hypothesis tests as independent-sample inference. The proposed CNN+GCN consistently outperforms the CNN baseline on Depth+Sobel inputs, with statistically significant improvements in PCK and mAP (p<0.001). The visibility-aware model showed a small but consistent improvement over CNN+GCN in the test set. To limit multiple-comparison bias, we restricted statistical testing to prespecified key comparisons.

### 4.3. Temporal Modeling

We selected the best-performing model architecture and input representation based on performance on the training and validation sets from the baseline dataset. We used validation performance, measured by mAP and PCK, to select the model architecture and input representation. We reserved the test set exclusively for the final evaluation of the selected models and did not use it for model or hyperparameter selection. This procedure avoided information leakage from the test set and provided an unbiased assessment of generalization performance. To incorporate temporal information, we explored a sequence-based architecture that combines convolutional feature extraction with a Long Short-Term Memory (LSTM) network on the baseline dataset. In this sequential approach, a CNN extracted pose from each frame, and an LSTM processed the resulting pose feature sequences to capture temporal dependencies in human motion and predict joint positions. We compared frame-wise and sequence-based pose estimation using a CNN-LSTM. This comparison evaluated whether temporal consistency improved pose estimation performance in continuous monitoring scenarios. We evaluated temporal modeling after frame-wise experiments to isolate the impact of temporal consistency from spatial input representations and backbone architectures.

Table 3 compares frame-wise pose estimation with sequence-based modeling using CNN+LSTM. The frame-wise model achieves the best overall performance, with a test performance score of 82.97% and a PCK of 76.45%. In contrast, the CNN+LSTM configuration shows a noticeable decline in performance, particularly in PCK (71.82%). In this experimental setting, temporal modeling with CNN+LSTM did not improve pose estimation performance. A likely reason is that depth-based frame inputs already provide strong spatial cues, while sequence modeling introduces temporal smoothing that may propagate localization errors across frames.

### 4.4. Real-World Scenario Evaluation

Finally, we evaluated the best-performing configurations under realistic deployment conditions to validate robustness in practical environments such as nursing homes. The evaluation included the following scenarios: single-person frames with partially visible joints, two-person frames, and frames of unoccupied rooms. This stage assessed the robustness and generalizability of the pose estimation framework in practical monitoring settings. These scenarios tested the models’ ability to handle partial visibility, multi-person scenes, and to correctly output no joint coordinates when no person is present.

For the two-person scenario, we used HRNet with a depth-based representation and L1 loss on the heatmap. We used HRNet for two people because, with multiple people in the frame, the simpler model failed to distinguish two skeletons. We therefore used a center heatmap to identify separate person centers and associate the predicted joint locations with the corresponding person. For partial-visibility and unoccupied-frame scenarios, we used depth with a Sobel representation with the CNN+GCN model. We also introduced a CNN with a visibility head to improve performance in separating visible and invisible joints. We used a shared convolutional feature extractor followed by a pose and visibility head that predicts the joints and visibility confidence, respectively. For real-world scenarios, we used the complete annotated ETAP-DID, with the split explained in Table 1 for the corresponding evaluation conditions; for example, for one person with partially visible joints, we used single-person data, and for an unoccupied room, we combined single-person data with unoccupied data.

#### Loss Function

In our real-world settings, the model estimated hallucinated values for invisible joints or estimated visible joints as invisible when we focused the loss function on visible joints. To force the model to learn both visible and invisible joints, we added more components to the loss function $L_{total}$ to improve robustness under occlusions, missing joints, and empty frames. The overall objective combines pose regression, visibility learning, structural consistency, and empty-scene regularization. Invisible joints are supervised using a mean squared error loss ($L_{inv}$), encouraging predictions toward a neutral origin when joint visibility is absent. A binary cross-entropy loss ($L_{vis}$) is used to learn joint visibility, enabling the model to distinguish between visible and occluded joints. For frames without a person, an empty-frame loss ($L_{empty}$) penalizes predicted joint activations and prevents hallucinated poses. The total loss function $L_{total}$ is used to train the pose estimation model, with all loss terms weighted and combined as follows:

(6) $$L_{total}=\lambda_{vis}L_{vis}+\lambda_{pose}L_{pose}+\lambda_{inv}L_{inv}+\lambda_{pck}L_{pck}+\lambda_{empty}L_{empty}$$

$\lambda_{vis},\lambda_{pose},\lambda_{inv},\lambda_{pck},\lambda_{empty}$ are hyperparameters that weight the contribution of each term and are set empirically during model development. We apply a person-presence mask to ensure pose-related gradients propagate only when a person is present, improving training stability. For the models without a visibility head, we set $\lambda_{vis}$ to zero. For the one person with missing joints scenario, we set $\lambda_{empty}$ to zero. During training, we normalized joint values to 0–1; during validation and testing, we converted them back to real coordinates.

Table 4 evaluates the best-performing configuration under realistic deployment conditions, including occlusions, multi-person scenes, and unoccupied-room scenarios. These experiments directly test the robustness of the proposed system in nursing home-like environments. We combined the first three cases (15 joints per person, including missing joints) into two-person and unoccupied frames for training, validation, and testing (see Section 2.2). In the last case, we added a baseline (7 all visible joints) with unoccupied frames for training, validation, and testing. Because the real-world experiments include a larger joint set and explicitly model missing joints, their PCK values are not directly comparable with those of the seven-joint baseline experiment.

The baseline+unoccupied configuration achieves the highest performance, with a test performance score of 84.63% and PCK of 67.51%. This indicates that explicitly incorporating unoccupied room conditions into the baseline dataset during training improves robustness. Performance decreases in more challenging scenarios. Single-person frames with missing joints yield a test PCK of 65.01%, reflecting difficulty in estimating both visible and invisible joints. The two-person scenario yields the lowest overall performance (PCK 64.52%), highlighting the ambiguity introduced by overlapping human structures in depth space. Unoccupied room conditions show relatively higher performance score but reduced PCK, indicating that while the model correctly identifies the absence of a person, residual predictions still affect joints. Using a visibility head slightly improves performance. Across all real-world scenarios, we evaluated statistical significance using two paired t-tests and Wilcoxon signed-rank tests over 500 test frames with PCK as explained before, comparing CNN+visibility against CNN+GCN. In the one-person scenario with missing joints, the improvement was statistically significant (p<0.01) with a small effect size (Cohen’s d≈−0.24). The negative sign of Cohen’s *d* reflects the direction of the subtraction and does not indicate a negative improvement. We observed similar significant improvements in the unoccupied-room and baseline+unoccupied settings (all p<0.001), providing evidence of a consistent improvement within the evaluated test set from the visibility-aware model.

### 4.5. Computational Analysis

Table 5 lists the hardware configuration used for the experiments, while Table 6 summarizes the computational requirements of the evaluated model. Among the lightweight architectures, CNN+GCN required the fewest computational resources: 3.74 GFLOPs, 3408 MB memory, 1.17 million parameters, and an average inference time of 2.6 ms per frame. Compared with the vanilla CNN, CNN+GCN reduced memory usage and parameter count and achieved faster inference. In contrast, ResNet18 and HRNet18 required substantially more memory and computational resources while providing lower pose-estimation performance on the baseline dataset. These measurements show that the CNN+GCN model has relatively low computational requirements compared with the higher-capacity architectures evaluated. However, since these measurements exclude preprocessing, data transfer, sensor I/O, and multi-room system overhead, they should not be interpreted as demonstrating end-to-end real-time deployment.

### 4.6. Discussion of Results and Limitations

The experimental results show that lightweight, structured representations provide the best balance of performance, robustness, and deployment efficiency for depth-based pose estimation. Among all evaluated configurations, the Depth+Sobel representation combined with CNN+GCN achieved the strongest overall performance, reaching 84.71% performance score and 77.18% PCK on the baseline dataset. Among the representations evaluated in the room-independent setting, Depth+Sobel combined with CNN+GCN achieved the highest test PCK of the compared configurations. This result suggests that the Sobel gradient representation may transfer well to unseen rooms within the evaluated dataset, although broader claims about cross-environment generalization require validation on additional sites and sensors.

In contrast, high-capacity architectures such as ResNet18 and HRNet18 produced lower generalization performance despite their higher capacity. High-capacity models tend to overfit; simpler models generalize better in noisy, real-world conditions [42]. The reduced performance of high-capacity models with Sobel inputs suggests that the additional gradient channels may introduce noise or redundant information when combined with these architectures.

These findings differ from prior pose estimation studies on RGB images, such as PopNet and PoseNet, where deeper architectures like ResNet50 improved localization performance by leveraging richer texture and appearance cues. Unlike RGB imagery, depth-only data contains limited texture and stronger sensor noise, making very deep models more prone to overfitting. The evaluated high-capacity architectures did not outperform lightweight models on this dataset. One possible explanation is that larger models were less well matched to the size, variability, and noise of the available training data. However, the present experiments do not directly establish overfitting as the cause of the performance difference. In practical healthcare environments, computational efficiency and robustness are critical. Therefore, lightweight CNN+GCN architectures offer a better trade-off between performance and hardware requirements than large-scale backbones such as ResNet50 or deeper HRNet variants.

The results also indicate that explicitly modeling skeletal structure is beneficial, as the CNN+GCN model consistently outperformed the vanilla CNN across all representations, improving the test performance score from 77.91% to 82.11% for depth-only input and achieving the best room-independent performance of 81.67% performance score and 76.03% PCK using Depth+Sobel input. For depth-based inputs, graph-based refinement improved performance in the room-independent setting, suggesting that explicit modeling of joint relationships can improve spatial consistency across unseen rooms.

Temporal modeling using CNN+LSTM did not improve performance. The frame-wise CNN model achieved an 82.97% test performance score and 76.45% PCK, whereas CNN+LSTM reduced PCK to 71.82%. Similar observations were reported in [19], where temporal smoothing did not improve motion continuity. In our setting, depth frames already provide sufficiently strong spatial cues, while temporal aggregation introduces accumulated errors and reduces responsiveness to abrupt pose changes.

Under realistic deployment conditions, performance degraded due to occlusions, overlapping body structures, and unoccupied-room scenarios. The baseline+unoccupied configuration achieved the highest robustness with an 84.63% test performance score and 67.51% PCK, demonstrating that explicitly incorporating unoccupied-room frames suppresses hallucinated poses. Performance decreased for single-person frames with missing joints (65.01% PCK) and was lowest for two-person scenes (64.52% PCK), where overlapping depth structures created severe joint ambiguity. Adding a visibility prediction head produced small improvements across the evaluated scenarios. These improvements were modest, indicating that visibility supervision provided incremental rather than transformational gains under the tested conditions.

CNN+GCN provided the best trade-off between localization performance and computational cost among the evaluated lightweight models. This assessment is based on its competitive localization performance and the lowest reported inference time and memory consumption. The graph-based component explicitly models joint relationships, which may improve spatial consistency while keeping computational requirements low. Overall, the results suggest that balancing accuracy, robustness, and deployment efficiency requires considering model complexity, computational cost, and performance under realistic conditions rather than maximizing model capacity alone. These findings support the practical use of lightweight CNN+GCN architectures for continuous monitoring in nursing home environments with limited hardware resources. However, this balance is specific to the evaluated dataset, sensor configuration, and hardware. Systematic validation across different environments and deployment conditions remains a limitation of the present study.

Limitations of this study include that the proposed framework still struggles in multi-person scenes due to depth ambiguity and lack of explicit person-separation mechanisms. Temporal consistency is not enforced beyond sequence modeling, limiting long-term tracking stability. Although lightweight models are better suited for deployment in nursing home environments, we conducted experiments on a relatively constrained ETAP-DID with limited subject diversity. We obtained the computational measurements on a single hardware configuration, which may not reflect performance on other CPU/GPU platforms or edge devices. In addition, we evaluated per-frame inference time and did not include the full sensor-to-decision pipeline or multi-room deployment overhead. The sensor sensitivity, response time, and the systematic optimization of mounting height and viewing angle were not independently evaluated. Finally, for partially visible participants, annotators manually estimated the locations of invisible joints. Since these inferred joint locations were not separated from visible-joint annotations in the evaluation, the reported performance may include uncertainty from manual estimation of occluded joints. Future work should consider a dedicated evaluation that separately analyzes visible and occluded joints to better characterize model performance under partial occlusion.

## 5. Conclusions and Future Work

This study evaluated depth-based 2D human pose estimation under realistic nursing home conditions, including partial visibility, multi-person scenes, and unoccupied rooms. Among the configurations tested, lightweight CNN-based approaches, especially CNN+GCN with Depth+Sobel input, offered a good balance of pose-localization performance and computational efficiency. However, this advantage should not be attributed to reduced overfitting because the experiments did not directly establish that mechanism. Performance decreased under partial visibility and was lowest in multi-person scenes. This indicates that depth ambiguity and incomplete observations remain key challenges. The visibility-aware model provided modest improvements. Temporal modeling with CNN+LSTM did not improve frame-level pose performance in the tested setup. Overall, the results support the feasibility of lightweight depth-based pose estimation for privacy-sensitive monitoring research.

### Future Work

Future work should address multi-person ambiguity by incorporating explicit instance-level separation mechanisms, such as detection-based pose grouping or transformer-based attention models. These approaches introduce technical challenges, including the need for larger annotated datasets and higher inference costs, particularly in real-time applications. Extending the framework to stronger temporal reasoning, potentially through transformer architectures or motion-aware graph networks, also presents challenges related to data scaling and longer training times. Such models typically require more memory and computational resources and may overfit when motion patterns are limited in the available training data. In our application, this is particularly challenging due to multi-person scenarios, noisy depth data, and limited annotated temporal sequences in realistic nursing home settings.

Another direction is enhancing robustness under adverse sensing conditions, including noise, missing depth regions, and sensor dropout. Future work will also explore adaptive joint selection strategies that dynamically identify the most informative joints based on the downstream task, although this may risk losing critical information for complex or rare events and requires careful validation.

Future work should also evaluate deployment on lower-power edge hardware and investigate model compression techniques such as pruning, quantization, and knowledge distillation. These approaches could reduce memory and inference requirements while maintaining sufficient pose estimation performance score for real-time monitoring.

## Figures and Tables

**Table 1:** ETAP-DID composition showing single-person, multi-person, and unoccupied durations (in hours), together with the number of rooms, across train, validation, and test splits.

Split	Single-Person		Multi-Person		Unoccupied		Total Duration
	Duration	Rooms	Duration	Rooms	Duration	Rooms	
Train	10.8 h	24	0 h	-	2.5 h	24	13.3 h
Validation	1.35 h	3	0.75 h	2	0.25 h	3	2.35 h
Test	1.35 h	3	0.75 h	2	0.25 h	3	2.35 h

**Table 2:** Performance comparison of input representations and models.

Input	Model	Val			Test		
		Acc ↑	PCK ↑	mAP ↑	Acc ↑	PCK ↑	mAP ↑
High-Capacity Architecture-Room-Dependent							
Depth	ResNet18	73.84	68.92	76.63	73.31	68.44	76.18
	HRNet18	**76.42**	**71.36**	**79.88**	**75.94**	**70.92**	**79.34**
Silhouette	ResNet18	70.42	65.61	74.84	70.34	64.72	73.94
	HRNet18	72.60	69.84	75.25	71.21	68.96	74.73
Depth+Sobel	ResNet18	72.91	68.11	75.84	72.36	67.74	75.31
	HRNet18	75.28	70.44	78.61	74.82	70.02	78.14
Lightweight Architecture-Room-Dependent							
Depth	CNN	78.24	73.18	81.46	77.91	72.84	81.02
	CNN+GCN	82.67	76.41	85.73	82.11	75.96	85.28
Silhouette	CNN	81.92	77.14	84.95	81.38	76.82	84.47
	CNN+GCN	79.84	74.62	82.76	79.21	74.05	82.11
Depth+Sobel	CNN	83.46	76.88	86.91	82.97	76.45	86.37
	CNN+GCN	**84.71**	**77.63**	**88.14**	**84.28**	**77.18**	**87.69**
Lightweight Architecture Room-Independent							
Depth	CNN	76.82	72.05	79.74	76.21	71.66	79.18
	CNN+GCN	79.18	74.11	82.06	78.63	73.74	81.58
Silhouette	CNN	79.42	75.28	82.47	78.96	74.91	81.96
	CNN+GCN	77.86	73.92	80.85	77.34	73.41	80.33
Depth+Sobel	CNN	80.94	75.82	84.16	80.31	75.27	83.61
	CNN+GCN	**82.13**	**76.48**	**85.42**	**81.67**	**76.03**	**84.95**

*Note:* ↑ indicates that higher values are better; bold values indicate the best performance within each corresponding input representation and architecture group.

**Table 3:** Frame-wise vs. sequence-based pose estimation on Depth+Sobel representation with room-dependent setting.

Method	Val			Test		
	Acc ↑	PCK ↑	mAP ↑	Acc ↑	PCK ↑	mAP ↑
Frame-wise (CNN)	83.46	76.88	86.91	82.97	76.45	86.37
Sequence (CNN + LSTM)	78.26	72.14	81.63	77.81	71.82	81.08

*Note:* ↑ indicates that higher values are better.

**Table 4:** Performance under real-world scenarios in a room-independent setting.

Scenario	Val			Test		
	Acc ↑	PCK ↑	mAP ↑	Acc ↑	PCK ↑	mAP ↑
One person + missing joints (CNN+GCN)	73.78	64.97	76.86	73.24	64.71	76.42
One person + missing joints (CNN+visibility)	74.18	65.27	77.36	73.64	65.01	76.92
Two persons (HRNet)	72.83	64.88	75.41	72.26	64.52	74.93
Unoccupied room (CNN+GCN)	81.04	66.88	83.62	80.51	66.54	83.17
Unoccupied room (CNN+visibility)	81.44	67.18	84.12	80.91	66.84	83.67
Baseline+unoccupied (CNN+GCN)	84.87	67.62	87.34	84.23	67.21	86.79
Baseline+unoccupied (CNN+visibility)	**85.27**	**67.92**	**87.84**	**84.63**	**67.51**	**87.29**

*Note:* ↑ indicates that higher values are better; bold values indicate the best performance within each corresponding input representation and architecture group.

**Table 5:** Hardware configuration used for inference experiments.

Component	Specification
GPU	NVIDIA GeForce RTX 2000 (12 GB VRAM)
CUDA Version	12.4
Processor	Intel(R) Xeon(R) CPU E5-2650 v4 @ 2.20 GHz
System Memory	256 GB RAM
Framework	PyTorch with GPU acceleration

**Table 6:** Hardware requirements comparison for input representations and models during the inference process.

Model	GFLOPs ↓	Memory (MB) ↓	Inf. Time (Avg) (s) ↓	Parameter Count
ResNet18	18.72	9875.00	0.0121	11,736,894
HRNet18	8.67	6122.00	0.0054	4,061,114
CNN	3.81	3824.00	0.0029	1,359,454
CNN+GCN	**3.74**	**3408.00**	**0.0026**	**1,169,250**
CNN+LSTM	3.87	4012.00	0.0032	1,926,268
CNN+visibility	3.83	3680.00	0.0028	1,358,293

*Note:* ↓ indicates that lower values are better; bold values indicate the lowest value among the evaluated models.

## Data Availability

The ETAP-DID cannot be shared due to the data protection and confidentiality requirements outlined in the approved study protocol and ethics agreement.

## Abbreviations

The following abbreviations are used in this manuscript:

AI	Artificial Intelligence
AP	Average Precision
CNN	Convolutional Neural Network
CNN+GCN	Convolutional Neural Network combined with Graph Convolutional Network
CNN+LSTM	Convolutional Neural Network combined with Long Short-Term Memory
CVAT	Computer Vision Annotation Tool
fov	field of view
fps	frame per second
GCN	Graph Convolutional Network
HAR	Human Activity Recognition
HMM	Hidden Markov Model
HRNet	High-Resolution Network
LSTM	Long Short-Term Memory
mAP	Mean Average Precision
PCK	Percentage of Correct Keypoints
RGB	Red, Green, Blue
RGB-D	Red, Green, Blue, Depth
ToF	Time-of-Flight

## References

[1] Viso Suite (2026). Popular Applications of Computer Vision in Site Security.

[2] X-Byte Enterprise Solutions (2025). Computer Vision Applications: How Industries from Healthcare to Retail Are Benefiting?.

[3] Stenum J., Cherry-Allen K.M., Pyles C.O., Reetzke R.D., Vignos M.F., Roemmich R.T. (2021). Applications of pose estimation in human health and performance across the lifespan. Sensors.

[4] Iyer H., Jeong H. (2026). A systematic review of computer vision-based human pose estimation for occupational safety. Int. J. Ind. Ergon..

[5] Radanliev P. (2025). Privacy, ethics, transparency, and accountability in AI systems for wearable devices. Front. Digit. Health.

[6] Javdan M., Ghasemaghaei M., Abouzahra M. (2023). Psychological barriers of using wearable devices by seniors: A mixed-methods study. Comput. Hum. Behav..

[7] Patel S., Park H., Bonato P., Chan L., Rodgers M. (2012). A review of wearable sensors and systems with application in rehabilitation. J. Neuroeng. Rehabil..

[8] Hu Y., Liu Y., Li H., Chen X., Yang Y. (2026). Battery capacity degradation trajectory prediction for wearable medical devices with deep temporal decomposition. Sci. Rep..

[9] Yao H., Ge C., Xue J., Zheng N. (2017). A high spatial resolution depth sensing method based on binocular structured light. Sensors.

[10] Luxonis ToF Depth Perception. https://docs.luxonis.com/hardware/platform/depth/ToF-depth.

[11] Dong C., Du G. (2024). An enhanced real-time human pose estimation method based on modified YOLOv8 framework. Sci. Rep..

[12] Lindroth H., Nalaie K., Raghu R., Ayala I.N., Busch C., Bhattacharyya A., Moreno Franco P., Diedrich D.A., Pickering B.W., Herasevich V. (2024). Applied artificial intelligence in healthcare: A review of computer vision technology application in hospital settings. J. Imaging.

[13] Prati A., Shan C., Wang K.I.K. (2019). Sensors, vision and networks: From video surveillance to activity recognition and health monitoring. J. Ambient. Intell. Smart Environ..

[14] Chou E., Tan M., Zou C., Guo M., Haque A., Milstein A., Fei-Fei L. (2018). Privacy-preserving action recognition for smart hospitals using low-resolution depth images. arXiv.

[15] Awan-Ur-Rahman, Borty S.C., Chakravarty S., Ramos M.D., Sakib N. (2026). Unpacking scopes & challenges in AI-driven health safety monitoring: A systematic literature review toward real-time fall and wandering monitoring for patients with dementia. Smart Health.

[16] D’Amato J.P. (2023). FitDepth: Fast and lite 16-bit depth image compression algorithm. EURASIP J. Image Video Process..

[17] Facil J.M., Ummenhofer B., Zhou H., Montesano L., Brox T., Civera J. (2019). CAM-Convs: Camera-aware multi-scale convolutions for single-view depth. Proceedings of the IEEE/CVF Conference on Computer Vision and Pattern Recognition.

[18] Hartmann Y., Klöckner J., Deichsel L., Paul R.E., Schultz T. (2024). Gait Parameter Estimation from a Single Privacy Preserving Depth Sensor. Proceedings of the 17th International Joint Conference on Biomedical Engineering Systems and Technologies-Volume 1: BIOSIGNALS. INSTICC.

[19] Hartmann Y., Paul R.E., Klöckner J., Deichsel L., Schultz T. (2025). Gait parameter estimation from a single depth sensor. J. Smart Cities Soc..

[20] Shahroudy A., Liu J., Ng T.T., Wang G. (2016). NTU RGB+D: A large scale dataset for 3D human activity analysis. Proceedings of the IEEE Conference on Computer Vision and Pattern Recognition.

[21] Sun K., Xiao B., Liu D., Wang J. (2019). Deep high-resolution representation learning for human pose estimation. Proceedings of the IEEE/CVF Conference on Computer Vision and Pattern Recognition.

[22] He K., Zhang X., Ren S., Sun J. (2016). Deep residual learning for image recognition. Proceedings of the IEEE Conference on Computer Vision and Pattern Recognition.

[23] Sun X., Shang J., Liang S., Wei Y. (2017). Compositional Human Pose Regression. Proceedings of the IEEE International Conference on Computer Vision.

[24] Cao Z., Hidalgo G., Simon T., Wei S.E., Sheikh Y. (2019). Openpose: Realtime multi-person 2D pose estimation using part affinity fields. IEEE Trans. Pattern Anal. Mach. Intell..

[25] Guo Y., Li Z., Li Z., Du X., Quan S., Xu Y. (2022). Pop-net: Pose over parts network for multi-person 3D pose estimation from a depth image. Proceedings of the IEEE/CVF Winter Conference on Applications of Computer Vision.

[26] Fredrik H. (2015). Privacy Protecting Surveillance: A Proof-of-Concept Demonstrator.

[27] Paul R.E., Devasia J.O., Kock P., Schultz T. (2026). Silhouette extraction from depth sensors for older adult monitoring at nursing homes. Proceedings of the Eighteenth International Conference on Machine Vision (ICMV 2025).

[28] Ding J., Wang Y., Yu L. (2010). Extraction of Human Body Skeleton Based on Silhouette Images. Proceedings of the Second International Workshop on Education Technology and Computer Science.

[29] Zhao S.L., Lee H.J. (2006). Human silhouette extraction based on HMM. Proceedings of the 18th International Conference on Pattern Recognition (ICPR’06).

[30] Dutta A., Lal R., Raychaudhuri D.S., Ta C.K., Roy-Chowdhury A.K. (2024). Poise: Pose guided human silhouette extraction under occlusions. Proceedings of the IEEE/CVF Winter Conference on Applications of Computer Vision.

[31] dos Reis E.S., Seewald L.A., Antunes R.S., Rodrigues V.F., da Rosa Righi R., da Costa C.A., da Silveira L.G., Eskofier B., Maier A., Horz T. (2021). Monocular multi-person pose estimation: A survey. Pattern Recognit..

[32] Paul R.E., Kock P., Hartmann Y., Ball E., Seibert K., Liu H., Schultz T. (2025). Longitudinal Data Acquisition for AI Services in Long-Term Care Facilities for Older Adults. Proceedings of the 18th International Joint Conference on Biomedical Engineering Systems and Technologies-Scale-IT-Up. INSTICC.

[33] CVAT.ai Corporation (2023). Computer Vision Annotation Tool (CVAT).

[34] Mejia-Trujillo J.D., Castano-Pino Y.J., Navarro A., Arango-Paredes J.D., Rincón D., Valderrama J., Munoz B., Orozco J.L. (2019). Kinect™ and Intel RealSense™ D435 comparison: A preliminary study for motion analysis. Proceedings of the 2019 IEEE International Conference on E-Health Networking, Application & Services (HealthCom).

[35] Moukthika Edge Detection Using OpenCV. 2025. Open Source Vision Foundation: 2025. https://opencv.org/blog/edge-detection-using-opencv/.

[36] Kim H., Sakamoto R., Kitahara I., Toriyama T., Kogure K. (2007). Robust silhouette extraction technique using background subtraction. Proceedings of the 10th Meeting on Image Recognition and Understanding (MIRU).

[37] Jalal A., Kim Y.H., Kim Y.J., Kamal S., Kim D. (2017). Robust human activity recognition from depth video using spatiotemporal multi-fused features. Pattern Recognit..

[38] Elhabian S.Y., El-Sayed K.M., Ahmed S.H. (2008). Moving object detection in spatial domain using background removal techniques-state-of-art. Recent Patents Comput. Sci..

[39] Schwenk K., Huber F. (2025). Connected Component Labeling Algorithm for very complex and high resolution images on an FPGA platform. Proceedings of the German Aerospace Center (DLR).

[40] Zheng C., Wu W., Chen C., Yang T., Zhu S., Shen J., Kehtarnavaz N., Shah M. (2023). Deep Learning-Based Human Pose Estimation: A Survey. ACM Comput. Surv..

[41] Henderson P., Ferrari V. (2016). End-to-end training of object class detectors for mean average precision. Proceedings of the Asian Conference on Computer Vision.

[42] Hermann K., Chen T., Kornblith S. (2020). The origins and prevalence of texture bias in convolutional neural networks. Adv. Neural Inf. Process. Syst..

